# Community Health Impacts From Natural Gas Pipeline Compressor Stations

**DOI:** 10.1029/2023GH000874

**Published:** 2023-10-31

**Authors:** Curtis D. Davis, Clara Frazier, Nihal Guennouni, Rachael King, Hannah Mast, Emily M. Plunkett, Zack J. Quirk

**Affiliations:** ^1^ Virginia Scientist‐Community Interface; ^2^ Department of Civil and Environmental Engineering University of Virginia Charlottesville VA USA; ^3^ Department of Biochemistry University of Wisconsin‐Madison Madison WI USA; ^4^ Virginia Institute of Marine Science William and Mary Williamsburg VA USA; ^5^ Department of Environmental Sciences University of Virginia Charlottesville VA USA; ^6^ Department of Chemistry Virginia Tech Blacksburg VA USA; ^7^ Department of Earth & Environmental Sciences University of Michigan Ann Arbor Ann Arbor MI USA

**Keywords:** natural gas infrastructure, air quality, community health, environmental justice

## Abstract

Compressor stations maintain pressure along natural gas pipelines to sustain gas flow. Unfortunately, they present human health concerns as they release chemical pollutants into the air, sometimes at levels higher than national air quality standards. Further, compressor stations are often placed in rural areas with higher levels of poverty and/or minority populations, contributing to environmental justice concerns. In this paper we investigate what chemical pollutants are emitted by compressor stations, the impacts of emitted pollutants on human health, and local community impacts. Based on the information gained from these examinations, we provide the following policy recommendations with the goal of minimizing harm to those affected by natural gas compressor stations: the Environmental Protection Agency (EPA) and relevant state agencies must increase air quality monitoring and data transparency; the EPA should direct more resources to monitoring programs specifically at compressor stations; the EPA should provide free indoor air quality monitoring to homes near compressor stations; the EPA needs to adjust its National Ambient Air Quality Standards to better protect communities and assess cumulative impacts; and decision‐makers at all levels must pursue meaningful involvement from potentially affected communities. We find there is substantial evidence of negative impacts to strongly support these recommendations.

## Key Points

- The impacts of compressor stations fall most heavily on the local communities around the stations, which are often socially vulnerable
- The current National Ambient Air Quality Standards are not sufficient to fully protect human health and vulnerable communities
- There is a severe need for more thorough air quality monitoring to understand the full impact of chemical emissions on community health

## 1 Introduction

### 1.1 What Are Compressor Stations, and Why do They Matter?

To maintain gas flow in natural gas pipelines, over 1,200 compressor stations pressurize natural gas every 50–100 miles along pipeline routes in the United States (Messersmith, 2015; U.S. EIA Office of Oil and Gas, 2007). We reviewed over 100 peer‐reviewed academic articles to synthesize a complete review of chemical emissions from compressor stations and the associated community health impacts. In this paper, we present a complete list of known pollutants emitted by compressors, evaluate the pollution in the context of currently available data and air quality standards, assess associated community impacts, and conclude with policy recommendations for state and federal agencies. Although necessary for natural gas pipelines, we find compressor stations significantly affect the well‐being of local communities and thus must be regulated accordingly.

Air pollution released by compressors is known to have significant negative health and environmental impacts to neighboring communities. Exhaust from combustion within compressor units is the major source of the air pollution, emitting chemicals that include volatile organic compounds (VOCs), nitrogen oxide compounds (NO_x_), and particulate matter (PM) (D. R. Brown et al., 2015; Green & Crouch, 2021; Hendryx & Luo, 2020; Johnson et al., 2015; Olaguer, 2012; Russo & Carpenter, 2017; van der A et al., 2020; Walter, 2020; White et al., 2019). Exposure to these air pollutants can be harmful to human respiratory, cardiovascular, and neurological systems and increase human mortality rates (Hendryx & Luo, 2020; WHO, 2021). Additionally, NO_x_ and VOCs react in the atmosphere to produce ozone, which aggravates human respiratory conditions like asthma (Grulke & Heath, 2020; U.S. EPA, 2020). While pollutants from compressor stations are widely known to be harmful to human health, there are few studies that directly link compressor station emissions to specific local community health outcomes (Green & Crouch, 2021; Hendryx & Luo, 2020).

Compressor stations are also significant sources of methane, a potent greenhouse gas and contributor to global warming (Strizhenok & Korelskiy, 2019). The majority of the methane is emitted during blowdowns, when compressor units are depressurized for maintenance and release large amounts of high‐pressure gas to the atmosphere (White et al., 2019). In the U.S. in 2020, compressors were estimated to have released 420,000 metric tons of methane, mainly during blowdowns, which is about 10% of the methane emitted from U.S. landfills in the same year (U.S. EPA, 2022b).

Although compressor station air pollution can be difficult to regulate, several federal laws apply to these emissions. Foremost, the Clean Air Act of 1970 (abbreviated CAA; see 42 U.S.C. §7401 et seq.) established National Ambient Air Quality Standards (NAAQS) to regulate air pollution from all sources, including compressor stations (U.S. EPA, 2021c). However, a lack of air quality monitoring near compressor stations has led to numerous violations of NAAQS in nearby communities (Babich, 2018). Compressor stations are also regulated under the National Emission Standards for Hazardous Air Pollutants as chemical emissions sources (Babich, 2018; Environmental Health Project, 2015). While there is a federal permitting process to build compressor stations, the EPA often delegates permitting to the state level. Many states and geographic areas have developed their own guidelines for compressor station permitting standards (Babich, 2018; Mountain Valley Pipeline LLC & Equitrans LP, 2017).

### 1.2 Compressor Stations and Environmental Justice

Decades of work by grassroot activists and academic researchers have documented the disproportionate placement of pollution‐generating infrastructure in historically marginalized communities, including the natural gas industry and compressor stations, which has resulted in high levels of air pollution exposure (Banzhaf et al., 2019; Collins et al., 2016; Emanuel et al., 2021; Mohai et al., 2009). These actions raise environmental justice (EJ) concerns and often result in frontline communities being subject to multiple pollution sources that compound to a higher cumulative exposure than if each source was considered alone.

In this paper, we use a definition of EJ that closely follows that of the Environmental Protection Agency (Environmental Justice, 2023), which defines EJ as: “the fair treatment and meaningful involvement of all people regardless of race, color, national origin, or income with respect to the development, implementation, and enforcement of environmental laws, regulations, and policies” (Environmental Justice, 2023). Fair treatment ensures no community bears “a disproportionate share of negative environmental consequences resulting from industrial, governmental and commercial operations or policies” (Environmental Justice, 2023). Meaningful involvement ensures people have an opportunity to participate in decisions that impact their lives and the decision‐making body is influenced by the public's voiced opinion (Environmental Justice, 2023). This definition includes the three core components of EJ: distributive (the distribution of environmental burdens), procedural (the policies and decisions that lead to the distribution), and recognitional (a sense of justice among stakeholders) (Banzhaf et al., 2019; Clough, 2018; Menton et al., 2020; Pearsall & Pierce, 2010; Rigolon et al., 2022; Svarstad et al., 2011).

Notably, compressor stations can contribute to all three EJ components. Emissions and associated human health outcomes contribute to distributive EJ based on where compressor stations are located. Federal and state policies that determine where compressor stations are sited can underlie procedural EJ issues; decision‐makers who create policy on fossil fuel infrastructure permits can make these decisions without considering the impacts on community members (Clough, 2018; Paparo, 2021). Although public engagement is required by many state and federal agencies, a lack of incorporation of public opinion into the decision‐making process can hinder recognitional EJ (Buckingham v. State Air Pollution Control Board & Atlantic Coast Pipeline, 6 VA. Ct. App. (No. 19‐1152), 2020; Daley & Reames, 2015; Wortzel & De Las Casas, 2021).

In addition to higher air pollution exposure, low income populations and communities of color are more likely to have underlying health conditions, driven mainly by social factors, that further increase susceptibility to environmental health hazards (Adler & Rehkopf, 2008; American Lung Association, 2023b; Murray et al., 2020). However, the interactions between pollution exposure, community vulnerability, and health outcomes remain under‐examined, and there are major unknowns regarding the long term and cumulative impacts of compressor stations on socially vulnerable communities that are less resilient when facing external stresses (ATSDR, 2022). Nevertheless, disproportionate placement of compressor stations in communities with EJ concerns is cause for alarm.

### 1.3 Scope of Paper

This paper addresses the knowledge gap between compressor station air pollution and specific local community health outcomes. We provide a review of major pollutants emitted by compressor stations and associated health impacts, and then evaluate how these emissions impact relevant policy, data quality, and community health. We conclude the paper with policy recommendations that aim to minimize the community health impacts from compressor stations.

## 2 Chemical Emissions From Compressor Stations: Specific Pollutants

It is well established that air pollution has negative health effects. Short‐term effects include symptoms such as headaches, nausea, and irritation of mucous membranes (WHO, 2021). In the long‐term, air pollution exposure is known to increase risk of lung cancer and cardiovascular and respiratory diseases (Thurston et al., 2017; WHO, 2021). Often, increased mortality rates can be directly attributed to higher air pollution exposure (Chen & Hoek, 2020; Hendryx & Luo, 2020; Murray et al., 2020; Orellano et al., 2020; WHO, 2021). Studies have also highlighted a variety of health issues affected by air pollution that may be less well‐known, including stroke, hypertension, diabetes, mental health effects, and negative reproductive and birth effects (Downey & van Willigen, 2005; Malin, 2020; Thurston et al., 2017; WHO, 2021).

Natural gas compressor stations emit a variety of airborne pollutants (D. R. Brown et al., 2015; Green & Crouch, 2021; Hendryx & Luo, 2020; Johnson et al., 2015; Olaguer, 2012; Russo & Carpenter, 2017; Strizhenok & Korelskiy, 2019; van der A et al., 2020; Walter, 2020; White et al., 2019). Compressor stations can have a significant effect on local air quality; in some rural environments, emissions from compressor stations can account for 98%–99% of VOC ozone precursors and 57%–61% of NO_x_ ozone precursors (Adgate et al., 2014). The main chemical emissions discussed below and highlighted in Table 1 are noteworthy because of their roles in two major forms of air pollution: smog and PM.

**Table 1:** Atmospheric Chemical Emissions From Natural Gas Compressor Stations, and Possible Health Effects and Regulatory Exposure Limits of Those Chemicals

Emissions	Health effects	Regulatory exposure limits
Nitrogen oxides (NO_x_), including nitric oxide (NO) and nitrogen dioxide (NO_2_)	Respiratory irritation and asthma; enhanced allergic responses.^a^ Ground‐level smog; reacts to produce ozone. Reacts to produce nitric acid;^b^ adverse respiratory effects when inhaled^c^	NAAQS primary standard: 53 ppb per year or 100 ppb per hour; secondary standard: 53 ppb per year.^d^ ^,^ ^e^ CAAQS: 0.18 ppm per hour or 0.030 ppm per year.^e^ ^,^ ^f^ WHO: 5.3 ppb per year or 13 ppb per day^e^ ^,^ ^g^
Ozone (O_3_)	Respiratory irritation, decreased lung function, and asthma;^c^ ^,^ ^g^ ^,^ ^h^ premature death notably linked to worsening of respiratory disease^g^ ^,^ ^h^	NAAQS primary and secondary standards: 0.070 ppm per 8 hr.^d^ CAAQS: 0.070 ppm per 8 hr or 0.09 ppm per hour.^f^ WHO: 0.05 ppm per 8 hr or 0.03 ppm in peak season^g^
Volatile organic compounds (VOCs)	Ground‐level smog; reacts to produce ozone. Reacts to produce secondary organic aerosols, a type of PM.^i^ Many have negative respiratory effects, are known or potential carcinogens, and can negatively affect neurological and/or cardiovascular systems^j^	N/A
Particulate matter smaller than 10 μm (PM_10_)	Respiratory irritation and asthma; cardiovascular diseases; lung cancer;^k^ ^,^ ^l^ increased respiratory, cardiovascular, and cerebrovascular mortality^k^ ^,^ ^m^ ^,^ ^n^	NAAQS primary and secondary standard: 150 μ g/m^3^ per day.^d^ CAAQS: 50 μ g/m^3^ per day or 20 μ g/m^3^ per year.^f^ WHO: 45 μ g/m^3^ per day or 15 μ g/m^3^ per year^g^
Particulate matter smaller than 2.5 μm (PM_2.5_)	Respiratory irritation and asthma; cardiovascular diseases; lung cancer;^k^ ^,^ ^l^ increased respiratory, cardiovascular, and cerebrovascular mortality^k^ ^,^ ^m^ ^,^ ^n^	NAAQS primary standard: 12.0 μ g/m^3^ per year; secondary standard: 15.0 μ g/m^3^ per year; or primary and secondary standards: 35 μ g/m^3^ per day.^d^ CAAQS: 12 μ g/m^3^ per year.^f^ WHO: 5 μ g/m^3^ per year or 15 μ g/m^3^ per day^g^

^a^ U.S. EPA (2016).

^b^ Seinfeld and Pandis (2016).

^c^ PubChem database, see Kim et al. (2021).

^d^ National Ambient Air Quality Standards (NAAQS), see U.S. EPA (2022d).

^e^ Standards are specifically for NO_2._.

^f^ California Ambient Air Quality Standards (CAAQS), see California Air Resources Board (2016).

^g^ World Health Organization (WHO) global air quality guidelines, see WHO (2021).

^h^ U.S. EPA (2020).

^i^ Qin et al. (2021).

^j^ Halios et al. (2022).

^k^ U.S. EPA (2019).

^l^ Chen and Hoek (2020).

^m^ Gray et al. (2015).

^n^ Orellano et al. (2020).

### 2.1 Atmospheric Smog

Ozone, a strong oxidant, is primarily responsible for the negative health effects associated with urban smog. Tropospheric ozone is formed through a series of photochemical reactions involving NO and NO_2_, collectively referred to as NO_x_. This photochemical pathway is the only significant source of ground‐level ozone (Baird & Cann, 2005; Seinfeld & Pandis, 2016); many regulations and research studies use concentrations of NO_x_ or NO_2_ as an indicator for overall severity of air pollution where ozone is a concern.

NO_x_ are consumed in radical reactions producing ozone, so local NO_x_ concentrations become depleted over a timescale of a few hours. When highly reactive gas‐phase VOCs are present, the VOCs also participate in photochemical reactions generating radicals and producing ozone, thus extending the lifetime of a smog event from a few hours to throughout the day (Baird & Cann, 2005).

### 2.2 Particulate Matter

PM encompasses a diverse group of atmospheric particles with a vast range of sources (biogenic and anthropogenic), chemical compositions, and sizes. The composition of PM is determined by its source. For example, particulates created from combustion, like those emitted by compressor stations, often have high levels of hazardous polyaromatic hydrocarbons (PAHs) (Baird & Cann, 2005; Lloyd & Cackette, 2001; U.S. EPA, 2019) and may also contain metals (Morajkar et al., 2020; Thiruvengadam et al., 2015; U.S. EPA, 2019).

PM_10_, particulates with an average diameter of 10 microns or less, are generally small enough to pass through the nose and throat and enter the lungs (Baird & Cann, 2005; Seinfeld & Pandis, 2016). With an average diameter of 2.5 microns or less, PM_2.5_ particulates are small enough to bypass bronchial cilia and other natural respiratory protections and interact directly with lung tissue (Baird & Cann, 2005). Because of this, PM_2.5_ is associated more strongly with negative health effects than PM_10_ (U.S. EPA, 2019) and thus is generally targeted more often in research and policy.

Due to the complexity of PM, it has proven difficult to isolate specific properties that contribute to or correlate with the most significant toxicity (Gray et al., 2015; Hime et al., 2018; WHO, 2021). Despite this, the broad effects of PM exposure are well‐understood and proven by decades of research (Chen & Hoek, 2020; Orellano et al., 2020; U.S. EPA, 2019; WHO, 2021). Notably, short‐term exposure, even on the time scale of hours to days, is associated with increased respiratory, cardiovascular, and cerebrovascular mortality (Gray et al., 2015; Orellano et al., 2020), likely representing deaths within the most vulnerable groups of the population. This is of particular importance given the tendency to place natural gas infrastructure in communities with EJ concerns (Emanuel et al., 2021).

## 3 Chemical Emissions From Compressor Stations: Evaluating the Context of Atmospheric and Health Data

### 3.1 Availability and Quality of Data

Prediction and quantification of health impacts from air pollution is complicated by many factors. Generally, a lack of data leads to challenges for establishing baseline levels of air composition and health factors in a community (D. Brown et al., 2014; Nathan et al., 2015). Potential chronic health effects require years of data to track and are therefore widely under‐examined (Hendryx & Luo, 2020). Point‐source emitters like compressor stations are also difficult to track with regards to accurate spatial and temporal fluctuations in pollutant concentrations. For instance, a study investigating air quality data from the Pennsylvania Department of Environmental Protection found that data averaged over long periods of time do not accurately capture short, high‐intensity chemical emissions events, such as compressor station blowdowns (D. R. Brown et al., 2015). Due to low sampling frequency and suboptimal siting, existing air quality monitoring may not always be representative of actual exposure for nearby community members (D. R. Brown et al., 2015; Transcontinental Gas Pipe Line Company LLC, 2019).

### 3.2 Degree of Exposure and Cumulative Health Impacts

A pollutant's mechanism of toxicity and degree of exposure are factors that affect the nature and severity of the pollutant's adverse health effects. The degree of exposure depends on the local concentration and atmospheric lifetime of a pollutant, two behaviors that are difficult to predict. Physical factors such as local geography and weather patterns can significantly impact pollutant concentrations (Baird & Cann, 2005; D. R. Brown et al., 2015; Mukerjee et al., 2019; WHO, 2021). For example, even the direction of wind can have additive or depleting effects on air pollution levels (Mukerjee et al., 2019).

The lifetime of an airborne pollutant also plays a role in the length of exposure. The atmospheric lifetime of a pollutant is terminated when the pollutant reacts to turn into a different chemical or deposits out of the atmosphere. Atmospheric reactions are typically complex and may vary considerably depending on the exact composition of the local chemical environment (Seinfeld & Pandis, 2016). In some cases, pollutants may undergo reactions to form other hazardous products. Additionally, PM can be created or modified when airborne chemicals aggregate, deposit onto an existing surface, or react with the chemical components of the PM (Seinfeld & Pandis, 2016). These factors affect the composition, lifetime, and fate of airborne pollutants, and are therefore important to consider when evaluating risks of chemical exposure.

There are many studies on negative health effects from exposure to one single type of pollutant or simple mixtures of common pollutants, but compressor stations emit more complex mixtures. A mixture of chemicals can change how pollutants are taken up by the body, as well as how fast the body can break them down (Löf & Johanson, 1998; Péry et al., 2013; WHO, 2021). This is particularly important because VOCs often react in the atmosphere and form different chemicals as secondary pollutants; when evaluating how a mixture of pollutants can change the severity of health effects, secondary pollutants also need to be considered.

Another complication is the possibility for compounding effects from other nearby polluting sources. Compressor stations are often located near other industrial units and infrastructure due to more convenient zoning and ease of access (Johns & Howell, 2016; Messersmith, 2015; Miles, 2016). These other infrastructure elements also contribute to airborne pollution (HEI Panel on the Health Effects of Traffic‐Related Air Pollution, 2010; Henneman et al., 2021; Lloyd & Cackette, 2001; Wang et al., 1999). Further, pollutants can travel up to hundreds of miles depending on geographic and weather conditions, such that rural communities can be exposed to significant amounts of urban pollutants (Baird & Cann, 2005; Mukerjee et al., 2019). It is apparent that the health risks of pollution combine, and may compound, with multiple exposures (Chestnut & Mills, 2005), so a complete risk analysis for a community must consider cumulative health risks.

### 3.3 National Ambient Air Quality Standards

Compressor stations are regulated by both federal and state laws. Although the EPA classifies a compressor station as a minor stationary emission source, numerous examples of NAAQS violations have been documented at compressor stations (Babich, 2018). This is often because the NAAQS specify different timescales of measurement for different pollutants depending on their lifetime in the atmosphere, but these measurement timescales may not be relevant for compressor stations that release a significant amount of emissions in a short time period, especially during blowdowns.

NAAQS are enforced through state‐dependent “state implementation plans” (SIPs). These SIPs are EPA‐approved documents that define each state's approach to ensure air quality is monitored and is compliant with the NAAQS (42 U.S.C. §7401 et seq.). Under the guidance of the EPA, each SIP outlines the requirements for sources of emissions to self‐monitor and self‐report controlled pollutants. Under this arrangement, the public must assume these sources will adequately monitor themselves. Although SIPs require self‐reported emissions data to be available to the public, it is possible that emitters may falsify or fail to report data, or report data that is unreliable due to poor measurement practices (Babich, 2018). Such concerns highlight the importance of total transparency in the process of collecting, reporting, and analyzing emissions data, as well as actively alerting the public of non‐compliance emissions events.

Furthermore, the NAAQS have faced criticism from experts (D. B. Brown & Rajan, 2022; Independent Particulate Matter Review Panel, 2020). For example, an independent EPA scientific advisory board expressed the need for tighter PM standards, claiming the current NAAQS for PM_2.5_ is not stringent enough to protect human health and emphasizing that any exposure to PM_2.5_ is harmful (Independent Particulate Matter Review Panel, 2020). Even the newly revised PM_2.5_ standards are not adequate (American Lung Association, 2023c; WHO, 2021). The American Lung Association also recently urged the EPA to lower its primary ozone standard, particularly emphasizing the health of people at higher risk (D. B. Brown & Rajan, 2022). Therefore, while there are federal regulations on compressor station emissions, we find that those regulations may not be sufficient to protect community health from negative health outcomes, especially for communities with EJ concerns where compounding factors often result in more severe negative effects (Adler & Rehkopf, 2008; American Lung Association, 2023b; Murray et al., 2020; Simoni et al., 2015).

### 3.4 Indoor Air Quality

On average, Americans spend 60%–95% of their time in their homes (U.S. BLS, 2020). Residential buildings are typically not well ventilated and often recycle air only 0.35 times per hour. This low level of air turnover can lead to an accumulation of pollutants from outside the home (ASHRAE, 2019; U.S. EPA, 2021a). Studies show that homes near compressor stations have VOC levels that exceed NAAQS, and that indoor VOC levels are often higher than levels measured just outside the homes (Caron‐Beaudoin et al., 2022; Martin et al., 2021). As suggested by current literature, we concur that current modeling of emission plumes and outdoor pollutants is not enough to ensure that compressor stations do not negatively affect the health of nearby residents, especially when considering the variability caused by weather events and on‐site activities (Martin et al., 2021; Payne et al., 2017). Further, Caron‐Beaudoin et al. (2022) point out that the environmental burden, and therefore the indoor air quality, of gas infrastructure lays heavily on communities with EJ concerns.

## 4 Community Impacts of Compressor Station Development

Many communities across the United States have felt the impacts of natural gas infrastructure, illustrating real world impacts of compressor stations on human health. The Marcellus Shale region of Pennsylvania is a hotspot for natural gas development. Within 10 years between 2008 and 2018, 15,939 natural gas wells were drilled in this region (Jacquet et al., 2018). As of 2019, Pennsylvania contained more than 500 compressor stations (Pennsylvania Department of Environmental Protection (PA DEP), 2019). The monetized damages associated with air pollution from natural gas extraction, including compressor stations, in Pennsylvania during 2011 has been estimated at $7,000,000–$32,000,000 (Litovitz et al., 2013).

The Marcellus Shale region has been a model for studies focusing on how compressor stations impact human health. During a study in Washington County PA, the U.S. Agency for Toxic Substances and Disease Registry (ATSDR) and the EPA identified nine pollutants as being above the recommended exposure limit, potentially affecting elderly or asthmatic persons (ATSDR, 2016). Other studies in the Marcellus Shale Region report that proximity to unconventional natural gas activity is associated with higher risk of heart failure (McAlexander, 2019), asthma (Rasmussen et al., 2016), depression and anxiety (Blinn et al., 2020), and disordered sleep (Casey et al., 2018). Despite strong links between natural gas development and disease, compressor station chemical emissions data remains sparse. In order to fully understand the impacts of compressor station emissions, air quality should be measured in more locations with increased frequency (Long et al., 2019).

Analyzing potential health impacts on surrounding communities is an important step in the natural gas development process, but these analyses are at risk of bias and inaccuracy. Mountain Valley Pipeline, LLC (MVP) planned to construct a new compressor station in Pittsylvania County VA, and commissioned Green Toxicology, LLC to conduct an air quality assessment. The MVP air quality permit was denied based on the report's failure to address EJ concerns according to the Virginia Environmental Justice Act (VA Air Pollution Control Board, 2021; Vogelsong, 2021). The report claims that PM from the compressor station would not aggravate asthma symptoms, despite clear evidence that PM irritates the respiratory system and can induce asthma (D. Brown et al., 2014; U.S. EPA, 2016; Volkodaeva & Kiselev, 2017).

Conflicts of interest in the consulting industry may lead to inadequate analyses of community impacts. One of the scientists from the MVP report has been challenged in the past on some of her claims (Wittenberg, 2021). While conflict of interest disclosures are required by the Federal Energy Regulatory Commission (FERC), they do not vet these disclosures, and other regulatory agencies may not have such requirements.

## 5 Policy Recommendations

The community health risks associated with living near a compressor station warrant stricter oversight by governments at the local, state and federal levels. Below, we describe several policy recommendations to limit air pollution exposure, assess community impacts, and increase transparency in the decision‐making process.

### 5.1 Increase Air Quality Monitoring and Data Transparency

Increased air quality monitoring at compressor stations is an utmost priority to address both acute and long‐term exposure effects. Although compressor stations are almost exclusively placed in rural areas, the EPA maintains most of their monitoring stations in urban areas (U.S. EPA, 2022a). Additionally, indoor air quality in homes near compressor stations is a concern (Martin et al., 2021). Natural gas suppliers in Pennsylvania, Ohio, and Louisiana have faced heavy fines for CAA violations at compressor stations, but a lack of consistent emission reporting makes it challenging to certify compliance at all sites (Russo & Carpenter, 2019; U.S. EPA, 2022c; Wright, Jr, 2022). Although emissions can be modeled, previous monitoring shows that where direct air quality measurements are taken, pollutant levels often exceed those produced by modeling techniques (Babich, 2018).

Ideally, monitoring should be continuous to capture the variability in air pollution emissions and blowdown periods when emissions are released in concentrated bursts. Without continuous monitoring, NAAQS violations will likely be missed in the data record (Babich, 2018). Continuous monitoring would also provide data for long term exposure studies, as prolonged exposure to lower levels of air pollution can also lead to negative health outcomes (Independent Particulate Matter Review Panel, 2020).

We recommend the EPA direct more resources toward monitoring programs at compressor stations and encourage states to incorporate monitoring into their CAA state implementation plans. Since natural gas infrastructure is concentrated in communities with EJ concerns, increased monitoring can also help federal and state agencies contribute to EJ initiatives such as Justice40 at the federal level (Emanuel et al., 2021; The White House, 2023). Pennsylvania has made recent progress in this area and can be a model for other states (Wolf & McDonnell, 2017). The EPA should also require that data collected in air quality monitoring near compressor stations is accessible and transparent to the public, ideally in easy‐to‐read maps with downloadable files containing the full data time series. There are existing tools, such as the EPA's AirData Air Quality Monitors application, but these tools should be comprehensive, including all data available to the EPA. We also recommend that the EPA conducts free indoor air quality tests upon request for homes that are within about 10 miles of a compressor station to make sure that community members are aware of health hazards they may face.

### 5.2 Stricter NAAQS

**Figure 1:**
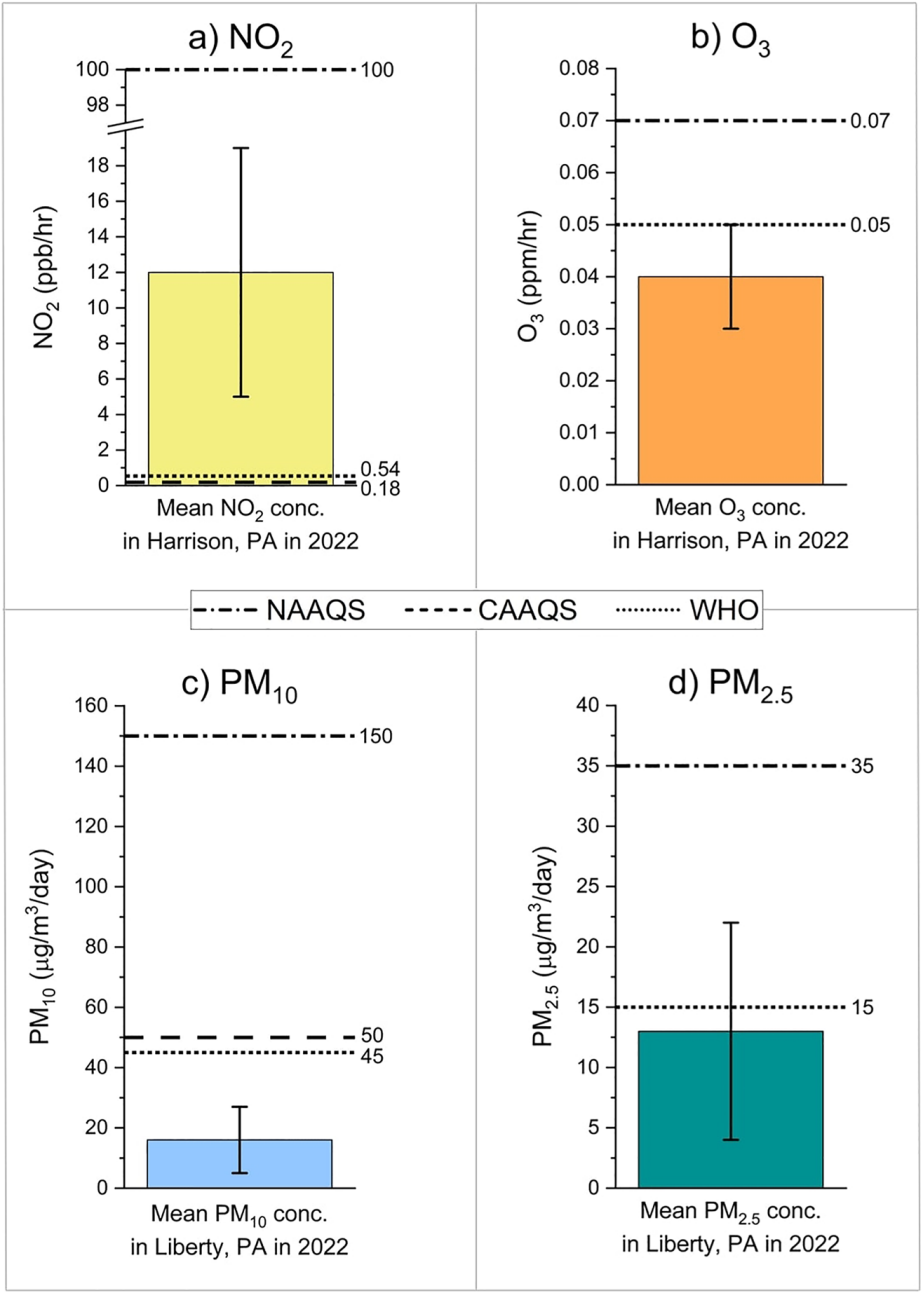
Mean pollutant concentrations are shown here to give a better understanding of the differences between the National Ambient Air Quality Standards (NAAQS), the California Ambient Air Quality Standards (CAAQS), and the World Health Organization (WHO) global air quality guidelines. Data shown are the arithmetic means and standard deviations of measurements taken throughout 2022, sourced from the EPA Air Quality System database. Both measurement locations are within 25 mi of Pittsburgh, PA in the Marcellus Shale region. Data from compressor station blowdowns are not readily available, but these activities increase one‐time concentrations significantly. Note that in (a), the WHO guideline for NO_2_ exposure per hour is estimated from the 24 hr guideline, and in (b), the CAAQS for O_3_ is the same as the NAAQS.

Even if monitoring improves and NAAQS are more consistently met by compressor stations, these standards do not adequately protect human health from exposure to air pollutants (American Lung Association, 2023a; American Lung Association et al., 2020; D. B. Brown & Rajan, 2022; Independent Particulate Matter Review Panel, 2020). Moving forward, air quality standards need to change to reflect the reality of cumulative exposures to air pollutants that many communities with EJ concerns face (Behles, 2011). We recommend that EPA adjusts the following standards to match the World Health Organization (WHO) (see Table 1 and Figure 1): NO_2_ (5.3 ppb per year; 13 ppb per day), O_3_ (0.03 ppm in peak season; 0.05 ppm per 8 hr), PM_2.5_ (5 μ g/m^3^ per year; 15 μ g/m^3^ per day) and PM_10_ (15 μ g/m^3^ per year; 45 μ g/m^3^ per day). We also recommend that NAAQS more strongly consider cumulative health impacts by evaluating a realistic mixture of air rather than a single pollutant on its own (Behles, 2011). Recognizing that changing the NAAQS is a challenging process, the EPA should at least incorporate cumulative impacts into its risk assessments. Cumulative impacts are often an issue in communities with EJ concerns, which makes proper evaluation all the more important.

### 5.3 Assess Community Impacts and Promote Community Engagement

Air pollutants often disproportionately impact communities with EJ concerns, yet air quality standards are not set with these communities in mind. Very little is understood about the cumulative impacts of exposure to air pollutants and regulations are developed assuming each exposure occurs independently when this is not often the case (Adgate et al., 2014). It appears that the Air Quality Index is the only metric that considers multiple exposure sources (U.S. EPA, 2021b). This index is based on regulation; it alerts residents of a particular area when one or all of the six core air pollutants exceeds recommended thresholds for human health (U.S. EPA, 2021b), but it is not related to control of industry emissions.

Community engagement has been recognized as a ladder of citizen participation, where at the lowest rung community members have little control over the decision‐making process (Arnstein, 1969). These lower levels of engagement allow for citizens to speak their views, but with little to no impact. Others have suggested more meaningful forms of engagement wherein the community actively works with the decision‐makers to reach a mutually beneficial agreement (Bidwell, 2016; Hagget, 2011). With this context in mind, we recommend that proposed compressor station activities require meaningful involvement from potentially affected community members. We define meaningful involvement based on the Virginia Environmental Justice Act (§2.2–234); this would require that decision‐makers actively seek out feedback from affected community members. Although meaningful involvement may take many forms, examples include community advisory boards or workshops for community members; these items are explored in more detail elsewhere (Hagget, 2011; Innes & Booher, 2007; Luyet et al., 2012). Any actions taken to improve community involvement should be thoroughly critiqued to ensure adequate citizen participation (Rowe et al., 2004). Community involvement will help to ensure that the community's needs are met during the development process, and that citizens are actively included in future decision‐making processes.

Digital rights and data transparency play important roles in ensuring meaningful community engagement in the decision‐making process. Although open government data initiatives have been deployed across the United States in order to increase emissions and air quality data transparency, many state governments may lack commitment to implementing these initiatives for EJ policymaking (Fusi et al., 2022). Although recent work in this area has focused on developing user friendly data visualization (Valencia et al., 2020) and setting guidelines for Indigenous data sovereignty (Carroll et al., 2020), the reality of data governance in the United States reveals underlying challenges that may hinder efforts to expand EJ policymaking (Dosemagen & Tyson, 2020; Dosemagen et al., 2022; Vera et al., 2019). As part of the effort to improve data transparency in the compressor station development process, we recommend that developers maintain data transparency regarding emissions and air quality during the review process and environmental data reporting. We suggest that data be published in an accessible manner that may be clearly understood by the affected communities. We recommend, however, that developers must respect the data sovereignty of any affected Indigenous groups, according to the CARE principles for Indigenous data governance (Carroll et al., 2020). To ensure complete transparency, we recommend that FERC vets conflict of interest disclosures to ensure their accuracy. We also recommend that contractors who have worked with pipeline companies in the past should be barred from working on FERC's behalf.

Virginia Department of Environmental Quality's (VA DEQ) Tidewater Air Monitoring Evaluation (TAME) Project provides an excellent example of fostering community involvement in the air monitoring process (VA DEQ, 2023). As part of this project, the VA DEQ deployed air monitors in two communities with EJ concerns in the Tidewater area to track how nearby coal storage and transportation affects air quality. Real time air quality data is available publicly online. The data collected during this project will be used by the VA Department of Health to communicate potential air quality risks to community members and develop strategies to combat health challenges. For these reasons, the TAME Project serves as a model for programs actively involving communities in the decision‐making process and using data transparency to facilitate public knowledge of health impacts.

It is crucial to ensure the community's health and wellbeing during compressor station development and planning. Implementing more stringent air quality standards and considering cumulative exposure risks will help to protect EJ communities that may face air quality concerns from a variety of sources. Community members should be well‐informed regarding potential health risks, have easy access to accurate air quality data, and have the opportunity to be an active part of the decision‐making process. With these changes, affected communities will have more power to protect citizens' health and advocate for their own needs.

## Conflict of Interest

The authors declare no conflicts of interest relevant to this study.

## Data Availability

The pollutant concentration data used for Figure 1 in the paper are available through the EPA's AirData website (see U.S. EPA, n.d.), which sources information primarily from the EPA's AQS (Air Quality System) database. On the AirData website, concentration data can be obtained through the interactive AirData Map App by selecting a specific air monitor of interest, or through downloading pre‐generated data files.
